# The Clinicopathological Features and Prognostic Significance of HER2-Low in Early Breast Tumors Patients Prognostic Comparison of HER-Low and HER2-Negative Breast Cancer Stratified by Hormone Receptor Status

**DOI:** 10.1155/2023/6621409

**Published:** 2023-11-30

**Authors:** Sanaa Gamrani, Laila Akhouayri, Sara Boukansa, Mehdi Karkouri, Hinde El Fatemi

**Affiliations:** ^1^Medical Center of Biomedical and Translational Research, Hassan II University Hospital, Faculty of Medicine and Pharmacy, University Sidi Mohamed Ben Abdellah, Fez, Morocco; ^2^Laboratory of Anatomic Pathology and Molecular Pathology, University Hospital Hassan II, Sidi Mohamed Ben Abdellah University, Fez, Morocco; ^3^Dipartimento di Scienze Cliniche e Biologiche, Università Degli Studi Di Torino, Via Giuseppe Verdi, Torino, Italy; ^4^Department of Biomedical Sciences, Genetics and Molecular Biology Laboratory, Faculty of Medicine and Pharmacy, Hassan II-Casablanca University, Rue Tariq Ibn Ziad, Casablanca, Morocco; ^5^Department of Pathology, Ibn Rochd University Hospital, Rue des Hôpitaux, Casablanca, Morocco

## Abstract

**Introduction:**

There has been increased interest in HER2-low breast tumors recently, as these tumors may have distinct clinical and molecular characteristics compared to HER2-negative and HER2-positive tumors. A new nomenclature has been proposed for HER2 1+ and HER2 2+ tumors that are confirmed negative according to fluorescence in situ hybridization (FISH). These tumors are now referred to as HER2-low, and it is thought that they may represent a distinct subtype of breast cancer that warrants further investigation. In this study, we aimed to evaluate the clinicopathological characteristics and prognostic impact of this particular subtype in a North-African context where HER2-low breast cancer is a relatively understudied subtype, particularly in non-Western populations.

**Methods:**

We conducted a retrospective cohort study on 1955 breast tumors in Moroccan patients over 10 years, collected at the Pathology Department of Ibn Rochd University Hospital in Casablanca and at the pathology department of Hassan II University Hospital in Fes. We elaborated on their complete immunohistochemical profile based on the main breast cancer biomarkers: Ki-67, HER2, estrogen, and progesterone receptors. Their overall survival and disease free survival data were also retrieved from their respective records.

**Results:**

Out of 1955 BC patients, 49.3% were classified as HER2-low; of which 80.7% and 19.2% were hormone receptors positive and negative, respectively. The clinicopathologic features indicate that HER2-low subtype tumors behave much more like HER2-positive than HER2-negative tumors. The survival analysis showed that the HER2-low subtype-belonging patients present significantly the poorest prognosis in disease-free survival (*p* = 0.003) in comparison with HER2-negative ones. When considering the hormonal status, hormonal-dependent tumors show a significant difference according to HER2 subtypes in disease-free survival (*p* < 0.001). Yet no significant difference was shown among hormonal negative tumors. Moreover, patients with hormonal positive tumors and simultaneously belonging to the HER2-low subgroup present a significantly good prognosis in overall survival compared to the ones with hormonal negative tumors (*p* = 0.008).

**Conclusion:**

Our study has shown that the HER2-low phenotype is common among hormone-positive patients. The clinicopathological features and prognostic data indicate that the hormonal receptors effect and HER2 heterogeneity are crucial factors to consider. It is important to note that this particular subgroup is different from the HER2-negative one and should not be treated in the same way. Therefore, this study offers a new perspective in the management of HER2-low patients and can serve as a basis for future prospective analyses.

## 1. Introduction

Breast cancer is a major global public health issue, as it is the most common cancer in women and the leading cause of cancer-related deaths [1]. Breast cancer is a complex and heterogeneous disease, which makes it difficult to diagnose and treat effectively [2]. Breast tumors are mainly categorized into five subtypes using the St. Galen surrogate based on the expression of biomarkers: estrogen receptor (ER), progesterone receptor (PgR), proliferation index (Ki-67), and human epidermal growth factor receptor 2 (HER2). Clinically, it is important to consider the prognosis and predictive impact of these biomarkers on breast tumors management [3, 4].

The HER2 gene (also known as c-erbB-2) is located on chromosome 17 q12-21.32. It belongs to the epidermal growth factor receptor (EGFR) family and plays a crucial role in cell communication through its tyrosine kinase activity. HER2 is a proto-oncogene responsible for regulating invasion, apoptosis, and proliferation [5]. Overexpression of the HER2 protein is the cause of HER2 gene amplification in roughly 15%–20% of breast cancer patients, and this is associated with a poor prognosis [6]. On the other hand, beyond its use for prognostic purposes, HER2 overexpression serves as selection criterion of patients who might be candidates for HER2-targeted therapies, such as trastuzumab monoclonal antibody [7]. Invasive primary or metastatic breast tumors with HER2 gene amplification or protein overexpression can be targeted by trastuzumab therapy, which binds to the extracellular domain and suppresses downstream signaling pathways, leading to improved outcomes [8, 9].

The assessment of HER2 protein expression is initially assured by immunohistochemistry (IHC), to evaluate the HER2 protein expression in the surface of the tumors cells using antibodies, but if the results are equivocal, fluorescence in situ hybridization (FISH) is recommended to reveal the HER2 gene copy number in the mammary cancerous cells [8, 10]. For the immunohistochemistry, the results are subsequently defined as a score ranging from 0+ (no or fewer than 10% of cells staining); 1+ (faint/barely perceptible membrane staining detected in >10% of the tumor cells); 2+ (HER2 is not uniform/low in intensity, but with circumferential distribution in at least 10% of cells, which makes it slightly equivocal, thus FISH is necessary to confirm its status); and 3+ (strong complete membrane staining of >10% of tumor cells) [11, 12].

In clinical practice, breast tumors are treated based on the expression of certain biomarkers. Tumors are classified as follows: The TNBC subtype is identified when there is no expression of the ER and PgR receptors, and when the HER2 score is 0 or +1 by IHC or +2 non-amplified by FISH test. The HER2+ subtype is identified when the staining is scored +3 or +2 and confirmed amplified by FISH. The Luminal subtype is identified when the ER and PgR expression is positive. receptors. It is important to note that over 83% of hormone-positive BC are HER2 negative (score 0 or +1 by IHC or +2 nonamplified by FISH) [13].

As mentioned earlier, drugs targeting HER2 revolutionized HER2-positive breast cancer treatment, creating remarkable survival outcomes for a once-gloomy BC subtype [14]. In the early stages of testing trastuzumab, the first anti-HER2 drug to be approved, it was found that only patients with tumors that stained 3+ for HER2 on immunohistochemistry (IHC) or stained 2+ but had HER2 gene amplification (2≥ two copies) on fluorescence in situ hybridization (FISH) showed positive responses to the treatment [15, 16]. Subsequent trials and international guidelines have used these early observations to establish the standard for testing and recommending anti-HER2 therapies [15]. While HER2-positive breast cancer only accounts for about 20% of new diagnoses, a greater proportion of patients (approximately 40–50%) have HER2 expression categorized as IHC 1+ or 2+ but FISH negative; moreover, this BC subgroup is classified with 0+ at IHC as HER2-negative for treatment decisions, excluding anti-HER2 therapies [14].

Actually, breast tumors with a HER2 score of 1+ by IHC and those scored 2+ and confirmed nonamplified by FISH are now proposed to be categorized as HER2-low. This new subtype of breast cancer may benefit from HER2-targeted therapy. For that, improving survival rates and developing precision medicine approaches for these patients are crucial. In this study, we aim to evaluate the clinicopathological characteristics and prognostic factors of patients with HER2-low breast carcinoma versus those with HER2-negative cancer based on HR profile in a North-African context. This study is the first of its kind in a North-African context and is based on a large dataset from two Moroccan centers.

## 2. Materials and Methods

### 2.1. Institutional Review Board Statement

The study design was analyzed and approved by the ethics committee of CHU Ibn Rochd of Casablanca, Morocco, under the following reference (No. 4/2022).

### 2.2. Patient Selection, Treatment

**Figure 1:**
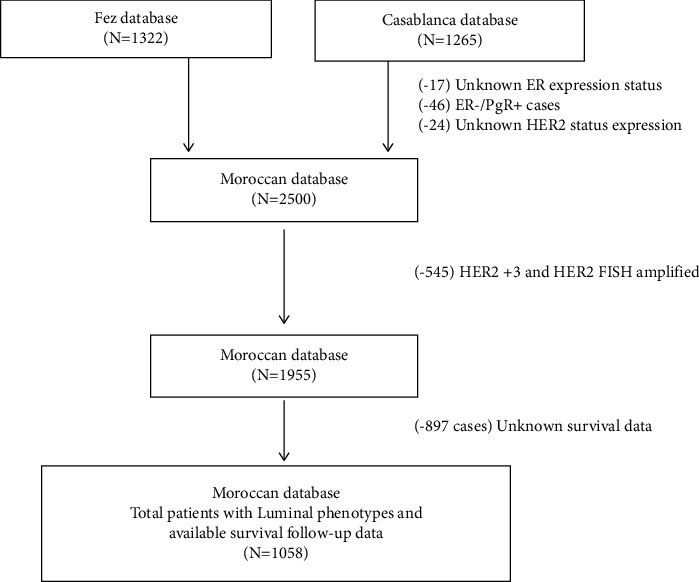
Study design and population.

This is a study that looked at 1322 cases of invasive breast cancer from the pathology department of Hassan II University Hospital in Fes and 1265 cases from the pathology department of Ibn Rochd University Hospital in Casablanca. The study was conducted over a 10-year period from 2012 to 2022. The epidemiological, clinical, and histological characteristics included age at diagnosis, SBR grade, tumor size (mm), lymph node infiltration, tumor stage, ER, PgR, HER2, and Ki-67 immunohistochemical status. The 10-year follow-up survival data were collected for all Fes patients, whereas it was only available and retrieved for 165 patients from the King Mohammed VI Center for Cancer Treatment in Casablanca (Figure 1). Specimens were obtained through biopsies in metastatic cases and through biopsies and surgical resections for nonmetastatic cases. Surgery was mainly radical mastectomy (Patey) or conservative surgery. All cases have been discussed in the multidisciplinary tumor board for deciding about neo-adjuvant treatment. All the decisions about radiotherapy, chemotherapy, hormone therapy, or targeted therapy conform to the European Society of Medical Oncology guidelines [15].

### 2.3. Data Collection

All invasive BC records were included. Contrastingly, benign tumors; tumors of uncertain malignancy; tumor recurrences; and patients with ER; PgR; and HER2 incomplete IHC and/or FISH status were all discarded.

#### 2.3.1. Definitions

HER2 status was defined as follows [17]:

(i) HER2-negative: if HER2 is confirmed negative (score 0) by IHC
(ii) HER2-low: if HER2 1+ (score 1) and HER2 2+ (score 2) are confirmed nonamplified by FISH. Subsequently, BC subtypes were described as being:
  1. HR+/HER2-low: for tumors with positive ER and/or PgR; and with HER2 1+and/or nonamplified HER2 2+ confirmed by FISH.
  2. HR+/HER2-negative: for tumors with positive ER and/or PgR; and with HER2 0+.
  3. HR-/HER2-low: for tumors with negative ER and/or PgR; and with HER2 1+ and/or nonamplified HER2 2+ confirmed by FISH.
  4. HR-/HER2-negative: for tumors with negative ER and/or PgR; and with HER2 0+.

#### 2.3.2. Histological Analysis

The histological analysis has been performed on formalin-fixed and paraffin-embedded (FFPE) tissue samples, stained with hematoxylin-eosin-saffron (HES). The assessment of histological classification and tumor grade was assured according to the Nottingham Histologic Score system. Nottingham histological grading score assigns a score of 1 to 3 for each parameter: degree of tubular formation, nuclear pleomorphism, and mitosis. The final histological grade is based on a sum of the individual scores of the three parameters: 3, 4, or 5 = Grade 1; 6 or 7 = Grade 2; and 8 or 9 = Grade 3 [18].

#### 2.3.3. Immunohistochemical Analysis

IHC analysis was performed on all primary invasive breast carcinomas using primary antibodies according to the manufacturer's guidelines. The staining was realized by BenchMARK ULTRA Ventana autoimmune stainer, with appropriate positive and negative controls including processing of normal tissue or tumor sections known to be positive. According to ASCO/CAP guidelines for 2020 [15], ER and PgR were considered positive if the tumor expressed at least 1% of nuclear staining cells, and HER2 was graded as negative (0, +1), equivocal (+2), and strong positive (+3) [19]. As for Ki-67, the cut-off was set to 20%. Subsequently, the results of the latter determined the percentage of immunoreactive cells over up to 2000 neoplastic cells. We have used the following primary antibodies: anti-Ki-67 (30-9, Ventana), antiestrogen receptor (SP1, Ventana, from 2011 to 2020), and antiprogesterone receptor (1E2, Ventana, from 2011 to 2020). For HER2, immunohistochemical analysis was carried out by using anti-HER2/neu (4B5, Ventana).

### 2.4. Fluorescence In Situ Hybridization Analysis

FISH analysis was conducted through PathVysion HER2 DNA Probe Kit (Abbott Vysis Inc., Downers Grove, IL) according to the manufacturer's instructions on 4 *μ*m thick sections of previously HER2 2+ scored samples by IHC. The probe mix included the LSI HER2/neu probe and the CEP17 probe. Fluorescence signals were analyzed by the CytoVision image analysis system in at least 20 counted tumor cell nuclei. A green signal in the nucleus identifies chromosome 17 diploidy; on the other hand, a red nuclear signal identifies the HER2 gene. HER2 (red)/CEP17 (green) ratio of 2 or more was recorded as positive amplification, while HER2/CEP17 ratios less than 2 was recorded as negative; according to (ASCO/CAP 2007 [20], 2013 [21], and 2018 [2]).

### 2.5. Statistical Analysis

The analysis was primarily conducted using the SPSS software, version 23.0. We evaluated the association between the HER2 status (HER2 0 and HER2 low) profile and other clinicopathological features using the chi-square test and Fisher's exact test as appropriate. The survival curves were performed using the Kaplan–Meier method and Cox regression to evaluate prognostic markers. We considered tests as statistically significant when *p* ≤ 0.05.

### 2.6. Follow-Up

Overall survival (OS) data reflects the difference in years between the first date of diagnosis and date of death or censoring whether alive or lost to follow-up. Disease-free survival (DFS) which reflects the difference in years between the first date of treatment and the date of local and/or distant recurrence diagnosis. In nonmetastatic breast cancer, there were regular follow-up examinations every 3-4 months for the first two years, every 6 months from year 3 to year 5, and then annually with an annual mammogram and regular bone density assessment for patients receiving AIs or ovarian suppression. In cases of metastatic breast cancer, follow-up was performed every 12 weeks with a clinical examination and a thoracic-abdominopelvic CT. The Kaplan–Meier method and the log-rank test were used to estimate and compare the laters.

## 3. Results

### 3.1. General Overview of the Clinic Pathological Features According to HER2-Low and HER2-Negative Subtypes Belonging

In this retrospective analysis, we focused on all patients with HER2 negative and HER2 low (patients with HER2 scored +2 and confirmed amplified by FISH and HER2 scored +3 by IHC were excluded), all patients were women with a mean age of 49 years, with the majority (53.1%) being older than 50 years. Most patients, up to 44%, were grade II, 28.3% of the rest were grade I, and then came 24.3% of patients who were grade III. Regarding TNM classification, 35% of patients are T2, 19.6% T1, and 8% T3. The majority of tumors are sized more than 2 cm. 48.1% presents positive lymph node metastasis. For the tumor stage, stage II was the most relevant with a percentage of 25%. The immunohistochemical results show positive expression for ER and PR and a high spread index in larger quantities of patients. When dividing the cases according to the HER2 status 50.7% were classified as HER2-negative, on the other hand, 49.3% were classified as HER2-low. The correlation between clinicopathological characteristics and HER2 status (low or negative) showed a significant statistical difference (*p* < 0.0001) in relation to SBR grade, tumor stage, tumor size, and lymph node involvement. The subgroup with HER2-low status had SBR grade I and tumor stage III, while the subgroup with HER2-negative status had SBR grade II and tumor stage II (Table 1).

### 3.2. The Clinicopathological Features of HER2-Low and HER2-Negative Subtypes in considering the HR Expression Status

When considering the hormone expression status, it was found that 49.3% of HER2-low tumors were hormone receptor (HR) positive (HER2-low/HR+), while 19.2% were hormone receptor negative (HER2-low/HR-). Significant differences were observed in SBR grade (*p* < 0.0001), tumor stage (*p* < 0.0001), tumor size (*p* < 0.0001), Ki-67 (HR-negative: *p* = 0.004, HR-positive: *p* < 0.0001), and lymph node involvement (HR negative: *p* < 0.0001, HR-positive: *p* = 0.004) when comparing the clinicopathological characteristics with HER2 subtypes in considering the HR status. The HER2-low/HR+ were more likely to be older at the time of diagnosis (52.9% were >50 yo), SBR histological grade I, tumor size T2, positive lymph node, and tumor stage III. Histological grade I was more frequent in terms of HR+/HER2-low tumors than HR-/HER2-low (41% and 28%, respectively) (Table 2).

### 3.3. Prognosis Implication of the HER2-Low Subtype Stratified by Hormone Receptors Status

#### 3.3.1. Overall Survival and Disease-Free Survival Rates According to HER2 Status in All Patients

**Figure 2:**
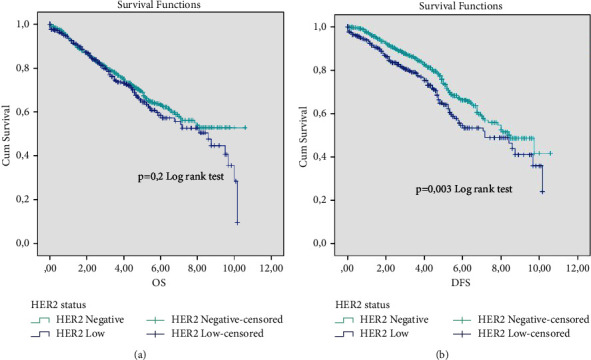
OS (a) and DFS (b) survival rates according to HER2 status in all patients.

Out of the 1955 enrolled patients, 1058 had available follow-up survival data. No significant difference was observed in terms of OS when comparing the HER2-low to HER2-negative subtypes, regardless of HR status membership (Figure 2(a)). In contrast, both subgroups differ significantly in DFS (*p*=0.003) in the overall follow-up period. With a 64% and 75% DFS rate for HER2-low and HER2-negative subtypes at 5 years of follow-up, which attributes a much poorer prognosis for patients belonging to the HER2-low subtype (Figure 2(b)).

#### 3.3.2. Overall Survival and Disease-Free Survival Rates of HER2-Low and HER2-Negative in HR+ Patients

**Figure 3:**
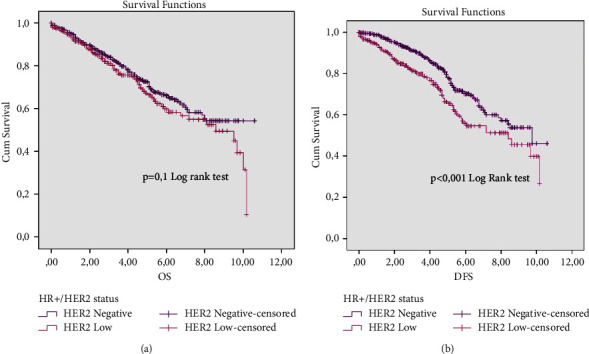
OS (a) and DFS (b) survival rates according to HER2 status in hormone positive.

Regarding the hormone-dependent tumors (HR+), they differ significantly in DFS (*p* < 0.001) with 66% and 80% as DFS rates for HR+/HER2-low and HR+/HER2-negative, respectively (Figure 3(b)). Yet no significant difference was spotted in terms of their respective OS rate (Figure 3(a)). In parallel, no significant difference was spotted in terms of HR tumors.

#### 3.3.3. Overall Survival and Disease-Free Survival Rates of HER2-Low Patients in considering the HR Status Expression

**Figure 4:**
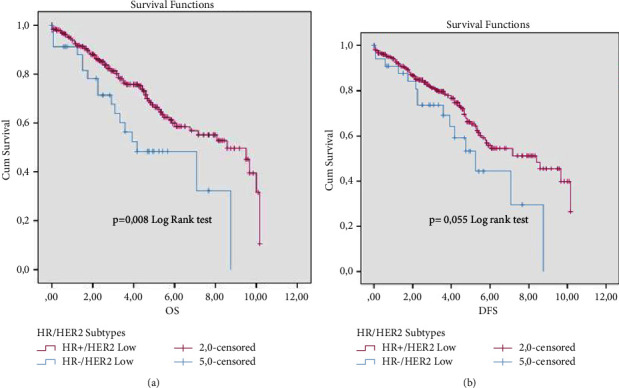
OS (a) and DFS (b) survival survival rates of HER2-low patients in considering the HR status expression.

Moreover, the HR+/HER2-low subgroup presents a significant and remarkable favorable prognosis in terms of OS (Figure 4(a)) in comparison to the HR-/HER2-low subgroup (*p*=0.008), with an OS rate of 50% and 70% for HR-/HER2-low and HR+/HER2-low at 5-years of follow-up, respectively. On the other hand, the difference was not significant in terms of DFS (*p*=0.055) (Figure 4(b)).

#### 3.3.4. Overall Survival and Disease-Free Survival Rates of HER2-Low and HER2-Negative Patients in Consideration of the Hormone Receptors Status

**Figure 5:**
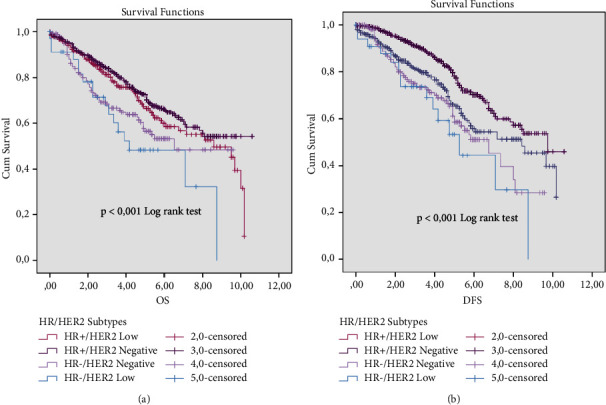
OS (a) and DFS (b) survival rates of HER2-low and HER2-negative patients in consideration of the hormone receptors status.

The patients' survival depending on their respective subgroups shows four significant OS curves (Figure5(a)) and DFS (Figure 5(b)) (*p* < 0.001). HR-/HER2-negative and HR-/HER2-low present the worst prognosis in comparison with the other subgroups, with a 5-year OS rate of 58% and 50%, respectively. Contrastingly, HR+/HER2-low and HR+/HER2-negative present a better prognosis impact, with a 5-year OS rate of 68% and 70%, respectively (Figure 5(a)).

## 4. Discussion

Recently, there has been an increased interest in the new classification of HER2 1+ and nonamplified HER2 2+ scores as HER2-low. However, this dichotomous classification has been challenged by emerging data on antibody-drug conjugate targeting HER2 among patients with metastatic HER2-low disease. Several recent studies suggest that HER2-low and HER-negative breast cancers may be different disease entities. A better understanding of this new subtype may open the opportunity for a large number of patients to benefit from HER2-targeted therapy. However, there is currently a lack of available data on the clinical distinction between both groups, as well as a lack of complete comprehension of the biology of low-HER2 breast cancer [22].

In the same perspective, the current study is the first and the largest real cohort to evaluate the clinicopathological and prognosis impact of HER2-low phenotype in an African context, and the Moroccan one in particular. According to previous studies and in line with our results the frequency of HER2-low phenotype ranges from 31% to 64% [23, 24]. Moreover, although the latter in our cohort was shown in both hormone-positive and hormone-negative tumors, the highest frequency goes to the positive ones. Similar results were reported previously by Denkert et al. [23] and Tan et al. [24], where HER2-low phenotype was found in almost 65% of hormone-positive tumors. On the other hand, Schettini et al. reported a greater expression of luminal genes in HER2-low tumors through PAM50 gene expression analysis [25]. This suggests that the expression of hormonal receptors and luminal genes may be the leading oncologic factor in HR+/HER2-low tumors [26].

In addition, we found that HER2-low tumors were more associated with higher Ki-67 scores and lymph node infiltration. In line with these results, Valentina et al. found that patients with HER2-low phenotype were more likely to present large-sized tumors, increased histopathological grade, higher Ki-67, and more common axillary lymph node involvement [27]. This suggests that the former behave more like HER2 positive tumors (HER2 3+) than HER2 negative ones (HER2 0+), although they are treated similarly [14].

In the perspective to assess the prognosis impact of HER2-low phenotype, compared to HER2-negative phenotype according to the tumor's hormone status, the difference was significant in terms of DFS, where HER2-low presented a poorer prognosis. This goes in line with a previous retrospective analysis elaborated by Valentina et al. who showed that tumors with moderate HER2 expression present an unfavorable prognosis in Luminal A, Luminal B/HER2 negative, and triple negative BC [27]. The same goes for Eggemann et al. who showed that the analysis of DFS based on HR status revealed that moderate HER2 expression was of an unfavorable outcome in HR-positive cases but not in HR-negative ones [28].

When considering HR expression, the only statistically significant difference was shown between HR+/HER2-low and HR+/HER2-negative in terms of patients' DFS. HER2-low tumors present the worst prognosis impact; this goes in line with another previous study by Tan et al. [24], yet in contrast with Agostinetto et al. findings, who showed that HER2 Low presents a favorable prognosis [26].

These results suggest that tumors with HER2-low phenotype are a heterogeneous group [29]. In our study, this heterogeneity was highlighted when comparing HR-/HER2-low with HR+/HER2-low subtype, which presented a good significant prognostic impact in overall survival and disease-free survival. A similar pattern was observed previously by Agostinetto et al. [14].

The current standard of care for breast cancer patients with HER2-positive disease involves the use of drugs that specifically target the HER2 receptor. which have been shown to improve outcomes in this patient population. Currently, a phase III trial is maintained, where patients with HER2-low tumors are treated with newly developed molecules called antibody-drug conjugates, the latter allows the use of HER2 as a vector of a cytotoxic drug. The use of this new treatment strategy shows promising results so far; in terms of the objective response rate (ORR), response duration, and progression-free survival (PFS) in the earlier phase I trial. In the future, the use of these new combined molecules may be the anticipated therapeutic strategy for this particular subtype [29–32].

The findings of our study have important implications for clinical practice, particularly in terms of how HER2-low breast tumors are classified and treated. Our study concludes that the introduction of HER2-low breast cancer has extended the benefits of anti-HER2 agents to more breast cancer patients. We found a higher frequency of the HER2-low phenotype in hormone-dependent tumors in a North African geographic context. In addition, our clinicopathological features and prognostic data underline the importance of the latter in HER2-low heterogeneity, indicating that this phenotype is distinct from the HER2-negative phenotype and should not be treated as such. It is important for clinicians to be aware of the potential differences between HER2-low and HER2-negative breast tumors and to consider the hormonal receptor status when making treatment decisions. Therefore, our study opens up a new perspective in the treatment management of a broad spectrum of HER2-low patients, which may serve as a basis for future prospective analyses that highlights the need for more research in this area.

## Figures and Tables

**Table 1:** General overview of the clinicopathological features according to HER2-low and HER2-negative subtypes belonging.

	HER2 status		
	HER2 low (%)	HER2 negative (%)	*p*
Age			0.3
<50yo	46.4	47.4	
>50yo	53.6	52.6	
SBR grade			**<0.001**
I	38.8	18.0	
II	38.2	49.8	
III	19.8	28.7	
NA	3.1	3.4	
Tumor size			**<0.001**
T0	0.0	0.5	
T1	17.4	21.8	
T2	29.4	41.6	
T3	8.0	7.8	
T4	1.3	7.3	
TX	43.8	21.1	
Lymph node			**<0.001**
N+	45.1	51.0	
N0	36.6	40.7	
NX	18.4	8.3	
Tumor stage			**<0.001**
I	8.7	10.9	
II	17.8	32.4	
III	20.0	28.3	
IV	1.0	3.0	
NA	52.4	25.4	
ER			0.2
Negative	19.2	20.3	
Positive	80.8	79.7	
PR			0.1
Negative	24.1	26.9	
Positive	75.9	72.9	
NA	0.0	0.2	
Ki67			**<0.001**
<20%	32.5	38.4	
>20%	57.5	45.6	
NA	10.0	16.0	

The values in bold presented in Table 1 indicate that the *p* value is less than 0.05.

**Table 2:** The clinicopathological features of HER2-low and HER2-negative subtypes in considering the HR expression status.

Clinicopathological characteristics	Hormone receptors negative			Hormone receptors positive		
	HER2 low (%)	HER2 negative (%)	*p*	HER2 low (%)	HER2 negative (%)	*p*
Age			0.3			0.5
<50yo	46.1	47.5		47.1	48.2	
>50yo	53.9	52.5		52.9	51.8	
SBR grade			**<0.001**			**<0.001**
I	41.2	22.5		28.1	1.0	
II	35.9	53.5		49.7	35.3	
III	19.3	20.8		20.5	59.7	
NA	3.6	3.1		1.6	4.0	
Tumor size			**<0.001**			**<0.001**
T0	0.0	0.4		0.0	1.0	
T1	18.6	23.4		13.0	14.9	
T2	30.9	40.7		24.9	46.8	
T3	7.4	6.9		10.3	10.9	
T4	1.3	6.7		1.6	10.0	
TX	41.7	21.9		50.3	16.4	
Lymph node			**<0.001**			**0.005**
N+	39.9	51.4		66.5	50.2	
N0	38.2	40.4		28.6	42.8	
NX	21.9	8.2		4.9	7.0	
Tumor stage			**0.001**			**<0.001**
I	9.6	11.6		4.9	7.5	
II	19.2	31.0		13.0	39.8	
III	19.6	28.8		22.7	26.9	
IV	1.1	2.8		1.1	4.0	
NA	50.6	25.7		58.4	21.9	
ER			—			—
Negative	—	—		100.0	100.0	
Positive	100.0	100.0		—	—	
PR			**0.005**			—
Negative	3.4	6.7		100.0	100.0	
Positive	96.6	93.1		—	—	
NA	0.0	0.3		—	—	
Ki67			**0.004**			**<0.001**
<20%	39.2	45.6		5.4	10.4	
>20%	49.5	41.1		90.8	62.7	
NA	11.2	13.4		3.8	26.9	

The values in bold presented in Table 2 indicate that the *p* value is less than 0.05.

## Data Availability

The data used to support the findings of this study are available from the corresponding author upon reasonable request.
